# Graphene optical modulators using bound states in the continuum

**DOI:** 10.1038/s41598-022-05253-4

**Published:** 2022-01-27

**Authors:** Myunghwan Kim, Sangin Kim, Soeun Kim

**Affiliations:** 1 Integrated Optics Laboratory, Advanced Photonics Research Institute, Gwangju Institute of Science and Technology, Gwangju, 61005 South Korea; 2 Department of Electrical and Computer Engineering, Ajou University, Suwon, 16499 South Korea

**Keywords:** Optoelectronic devices and components, Photonic crystals

## Abstract

Graphene-based optical modulators have been widely investigated due to the high mobility and tunable permittivity of graphene. However, achieving a high modulation depth with a low insertion loss is challenging owing to low graphene-light interaction. To date, only waveguide-type modulators have been extensively studied to improve light-graphene interaction, and few free-space type modulators have been demonstrated in the optical communication wavelength range. In this study, we propose two graphene-based optical free-space type modulators in a simple silicon photonic crystal structure that supports bound states in the continuum. The designed modulator with an ultra-high quality factor from the bound states in the continuum achieves a high modulation depth (MD = 0.9972) and low insertion loss (IL = 0.0034) with a small Fermi level change at the optical communication wavelength. In addition, the proposed modulators support outstanding modulation performance in the normal chemical vapor deposition (CVD) graphene (mobility = 0.5 m^2^/Vs). We believe the scheme may pave the way for graphene-based optical active devices.

## Introduction

Graphene, where a single layer of atoms is arranged in a two-dimensional honeycomb lattice, has gained considerable attentions owing to its various exceptional properties, such as its high thermal conductivity, ultra-high saturable absorption, and wide optical bandwidth^1–3^. Numerous graphene-based optical devices have been studied for many years; these include photo detectors^4–7^, optical absorbers^8–11^, and nonlinear devices^12–15^. In particular, graphene-based optical modulators, one of the key components of photonics systems, have been extensively investigated. The outstanding carrier mobility and gate-tunable carrier concentration of graphene enable it to be used as an active medium in optical modulators; the carrier concentrations of graphene can be tuned by applying different gate-voltage (Fermi level variation), which enables gate-tunable absorption of graphene^16^.

To date, many waveguide and free-space optical modulators using graphene loss variation have been proposed over a wide wavelength range. Optical transitions, including interband and intraband transitions, are the main processes that determine the loss of graphene. In the terahertz and mid-infrared wavelength regions, where intraband transition is dominant, absorption in graphene is largely tuned by adjusting the drive voltage. Therefore, many highly efficient graphene-based optical modulators have been demonstrated in these wavelength regions, such as optical modulators using metal reflectors^17^, graphene metamaterials^18–21^, graphene antennas^22^. In contrast, graphene loss is largely determined by the interband transition in the optical communication wavelength region, which is nearly independent of the Fermi level of graphene. Therefore, waveguide-type modulators have been extensively studied to improve light-graphene interaction in this wavelength range. A graphene-coated Si waveguide-type optical modulator operating in the optical communication wavelength was the first to be experimentally demonstrated^23^. This device had a small device area (~ 25 μm^2^) with a high modulation depth (MD = 0.1 dB/μm), compared to conventional Si-based optical modulators. Subsequently, double graphene layer-coated waveguide-type optical modulators^24,25^, suspended graphene modulators^26^, and hybrid graphene modulators^27^ have been demonstrated with improved the modulator performance. However, waveguide-type modulators have a limited range of applications because they exhibit a trade-off between modulation depth and insertion loss (IL). In particular, they are not suitable for free-space applications that require a low insertion loss. Lee et al. proposed a free-space graphene modulator composed of a quarter-wavelength-thick insulator layer and a metal reflector to increase graphene absorption by placing the graphene layer where the amplitude of the electric field was maximized^28^. However, this modulator showed a very low modulation depth of 4% owing to the low absorption of graphene (2.3% absorption for normal incidence).

To increase absorption in graphene, optical modulators using the epsilon-near-zero effect have been introduced^29,30^. These devices achieved a high modulation depth with an extremely enhanced electric field in the graphene layer. However, the epsilon-near-zero effect in graphene is highly debated and has not been experimentally demonstrated. Another approach to increase light-graphene interaction is to place a graphene layer near high Q-factor resonators, such as photonic crystal resonators^31^ and whispering gallery modes^32,33^. However, these resonators show relatively low Q values (Q = 10^3^ ~ 10^4^), which is not enough to achieve highly efficient modulators; Scattering loss by imperfect fabrication and graphene loss deteriorate the Q value.

In this study, we propose two graphene-based optical modulators using bound states in the continuum (BICs) in a simple one-dimensional photonic crystal structure. Theoretically, BICs support infinite Q resonance as a result of destructive interference between radiative waves, and they can be easily obtained from photonic crystal structures^34–40^. In addition, high Q (> 10^5^) is maintained in the symmetry broken structures due to imperfect fabrication: tiled, imperfectly etched, or bent structure^34^. In the proposed scheme, the high Q transmission resonance from the BIC facilitates a sharp transmission variation from the tuning of the Fermi level of graphene, which enables the proposed modulators to simultaneously achieve a very high modulation depth and low insertion loss. We also investigate the effect of graphene mobility on modulation efficiency. The proposed modulators maintain high performance if the graphene mobility is higher than *μ* = 0.5 m^2^/Vs, and this value can be obtained from normal graphene.

## Results

### Structure of the modulators

**Figure 1:**
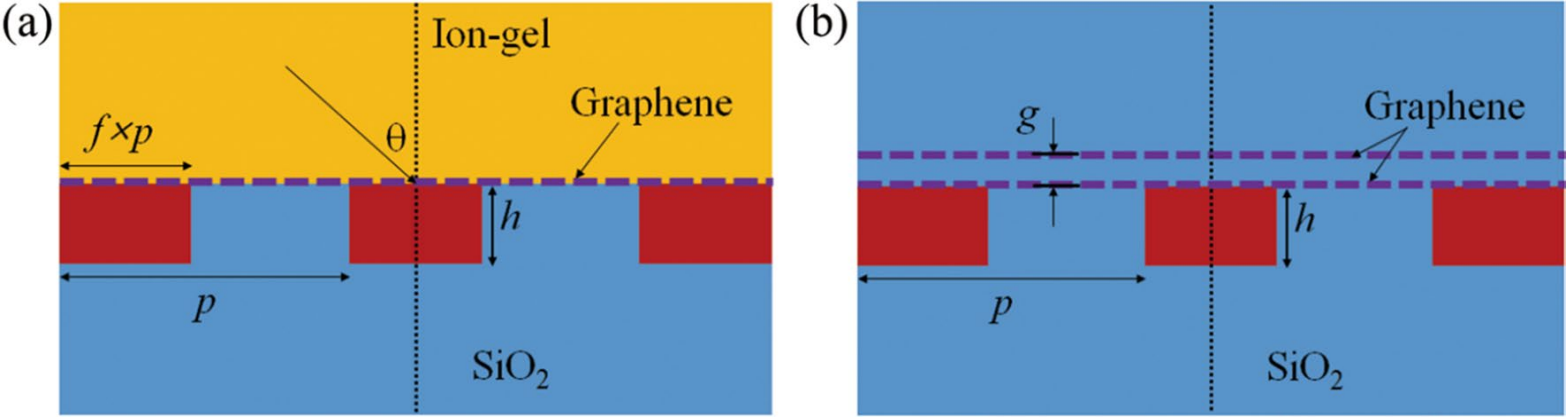
Schematic diagrams of the graphene-based optical modulators. (**a**) Ion gel and single-layer graphene modulator and (**b**) double-layer graphene modulator.

Figure 1a,b show schematics of the proposed optical modulators composed of ion gel and single-layer graphene (ISLG), and double-layer graphene (DLG), respectively. In the figures, *p* is the period, *f* is the fill factor, *h* is the height of Si, and *θ* is the incident angle. The refractive indices of Si, ion gel, and SiO_2_ are 3.45, 1.45 and 1.45, respectively. The permittivity of graphene is calculated from the Kubo formula^41^, and the height of the graphene layer is assumed to be 0.34 nm. Ion gel is used for the electrical doping of graphene in the ISLG modulator structure. Note that there are many kinds of ion-gel materials. The refractive index of most of the ion gels is in the range of 1.4 ~ 1.45, and IL-P14 and IL-AP3 have the refractive index of 1.45^42–44^. In recent years, ion-gel materials have been actively utilized as an efficient gating medium. They have great advantages such as transparent, good mechanical flexibility, thermal stability, easy fabrication, and compatibility with various substrates. The electric double layer with extremely high capacitance at the graphene-ion gel interface enables the chemical potential (Fermi level) of graphene to be adjusted with a low electric gate voltage^45–47^. In the DLG structure, graphene doping is implemented by applying a gate voltage between two graphene layers.

**Figure 2:**
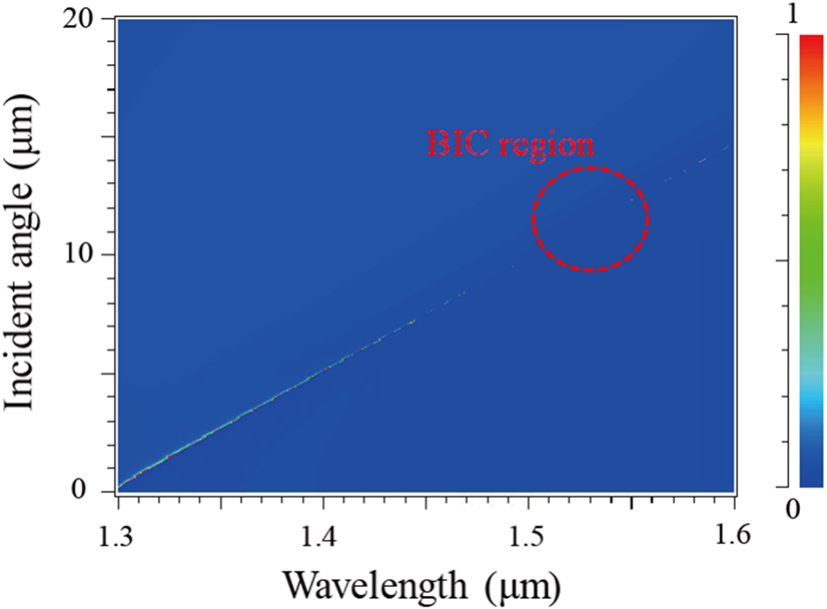
Reflection spectrum as a function of the incident angle: *p* = 870 nm, *h* = 150 nm, and *f* = 0.3.

First, we designed a photonic crystal structure to induce a BIC phenomenon. Figure 2 shows the reflection spectrum as a function of the incident angle in the proposed photonic crystal structure without graphene layers. We used the following parameters: *p* = 870 nm, *h* = 150 nm, and *f* = 0.3. The parameters were selected to induce the BIC phenomenon near the operating wavelength. The BIC phenomenon is observed near λ = 1.54 μm and *θ* = 12°, and the reflection peak (transmission dip) disappears in this region due to the infinite Q because of BIC.

**Figure 3:**
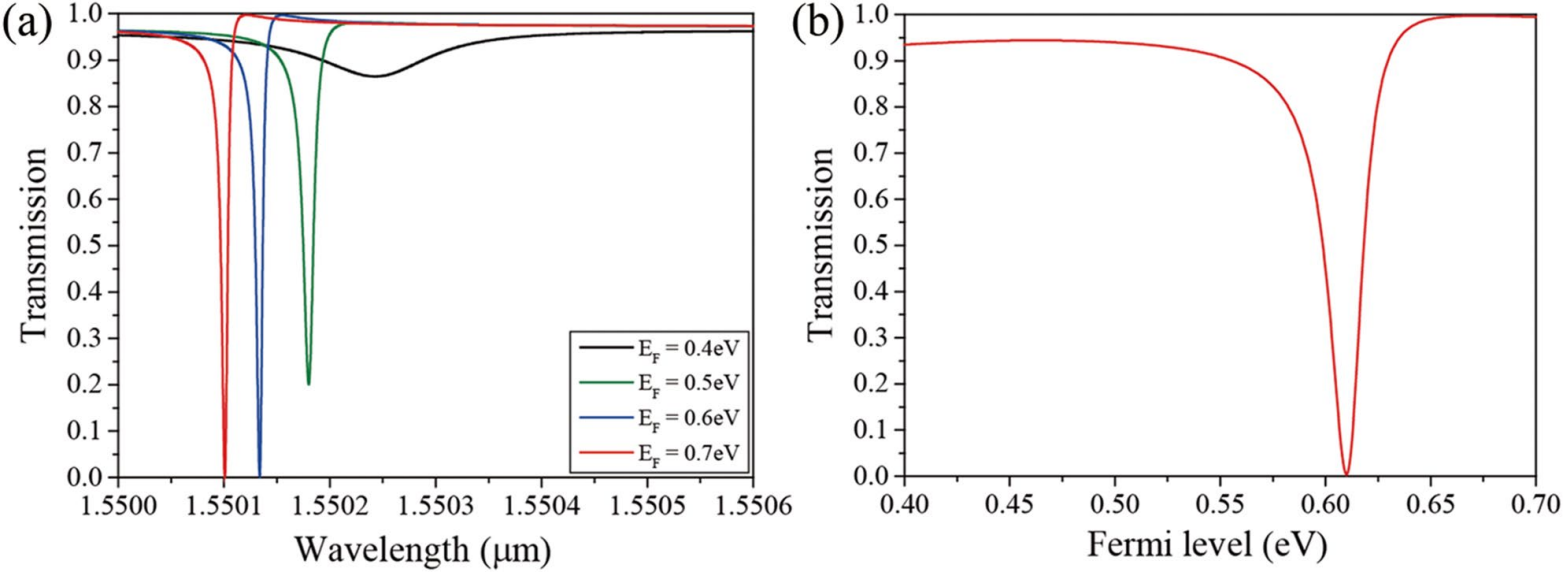
(**a**) Transmission spectra for Fermi level variation from E_F_ = 0.4 eV to 0.7 eV. (**b**) Transmission variation as a function of the Fermi level in the SLG modulator. The mobility of graphene is *μ* = 1 m^2^/Vs.

We investigated the variation in the transmission spectra for Fermi level variation in the proposed ISLG structure. The mobility of graphene is assumed to be *μ* = 1 m^2^/Vs. In this case, the BIC disappears owing to the graphene loss; Q is inversely proportional to the loss, and the loss of graphene breaks the infinite Q condition of the BIC. However, an ultra-high Q is maintained because the loss of doped graphene is insignificant, which facilitates the design of high-performance modulators. Figure 3a shows the transmission spectra of the ISLG for the Fermi level variation from E_F_ = 0.4 eV to 0.7 eV at the incident angle of *θ* = 11.937°. By choosing the incident angle slightly apart from the BIC, the transmission resonance with ultra-high Q at the operating wavelength can be achieved. The other parameters are assumed to be the same as in the previous calculations. As increase in the Fermi level leads to a decrease in the graphene loss, the Q of the transmission dip increases with an increase in the Fermi level. The Q of the resonance for E_F_ = 0.7 eV is Q ~ 2 × 10^5^, and it can be simply increased by adjusting the incident angle. In addition, it has been shown that increasing the Fermi level of graphene leads to a transmission dip blue shift because the permittivity of graphene decreases as the Fermi level increases. Generally, graphene doping (Fermi level variation) has little effect on tuning the resonant wavelength in resonators because the variation of the graphene permittivity does not significantly change the effective index of the structure due to the very thin graphene. However, in this ultra-high Q resonance system, a small resonant wavelength tuning results in a very high transmission variation. The transmission variation for the Fermi level variation at λ = 1.55013 μm is shown in Fig. 3b. The transmission becomes approximately zero and one at E_F_ = 0.615 (off-state) and E_F_ = 0.672 eV (on-state), respectively. Therefore, a very high modulation efficiency was achieved: MD = 0.9972 and IL = 0.0034, where the modulation depth was obtained as MD = (T_on_-T_off_)/T_on_, and insertion loss, which is ratio of the transmitted power for on-state and input power, was calculated as IL = 1 − T_on_.

**Figure 4:**
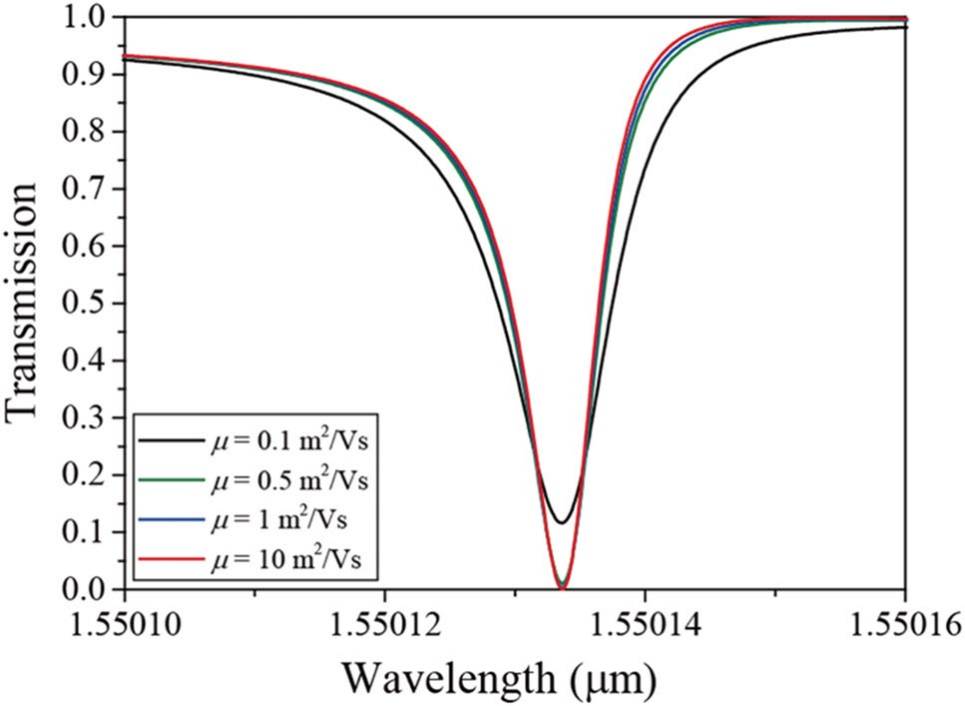
Transmission spectra for graphene mobility variation from *μ* = 0.1 to *μ* = 10 m^2^/Vs for E_F_ = 0.6 eV.

Figure 4 shows the transmission spectra for the mobility variation from *μ* = 0.1 m^2^/Vs to *μ* = 10 m^2^/Vs for E_F_ = 0.6 eV. Because the mobility is inversely proportional to the loss, a higher Q is observed for a higher graphene mobility. However, the high Q is maintained even though the mobility is *μ* = 0.1 m^2^/Vs, and the transmission spectra for *μ* > 0.5 m^2^/Vs are almost the same. Therefore, a high modulation efficiency can be sustained for *μ* > 0.5 m^2^/Vs. Note that the real part of graphene permittivity is almost constant for the mobility variation. Therefore, the resonant wavelength hardly shifts for the mobility variation. In addition, as the mobility increases, the imaginary part of the graphene permittivity sharply decreases in the range of *μ* = 0 ~ 0.5 m^2^/Vs. Therefore, the transmission spectra largely change when the mobility is varied from *μ* = 0.1 to 0.5 m^2^/Vs. It should be noted that a mobility of *μ* = 0.5 m^2^/Vs can be obtained from the chemical vapor deposition (CVD) method that is considered the most promising method for producing graphene^48,49^.

**Figure 5:**
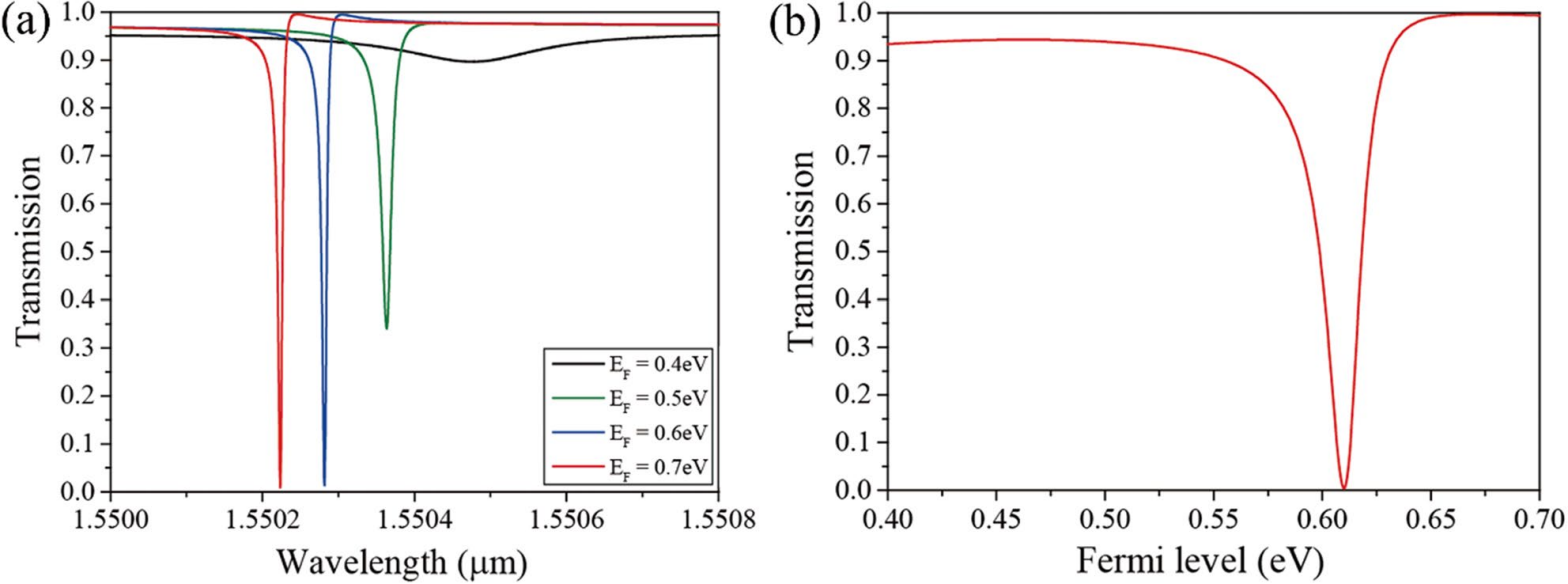
(**a**) Transmission spectra for Fermi level variation from E_F_ = 0.4 eV to 0.7 eV. (**b**) Transmission variation as a function of the Fermi level in the DLG modulator. The mobility of graphene is *μ* = 1 m^2^/Vs.

Although the ion gel-based modulator shows outstanding modulation performance, the low stability and slow modulation speed of the ion gel are obstacles to realizing high-speed optical modulators. To solve these problems, we designed an optical modulator composed of two graphene layers (Fig. 1b). By applying a gate voltage between the two graphene layers, stable high-speed graphene doping is possible. Figure 5a shows the transmission spectra for the Fermi level variation. The mobility of graphene is assumed to be *μ* = 1 m^2^/Vs. The Q of the resonance is slightly reduced compared to that of ISLG owing to the multiplied graphene loss. However, this modulator also supports a very high Q transmission. The transmission variation for the Fermi level at an operating wavelength of λ = 1.55028 μm is illustrated in Fig. 5b. The calculated modulation depth and insertion loss are approximately MD = 0.9889 and IL = 0.011, respectively. The modulation depth was slightly reduced, and the insertion loss slightly increased. However, very high modulation performances are maintained.

## Conclusions

In this study, we proposed two graphene-based free-space high-performance optical modulators with a simple photonic crystal structure. The very high Q transmission resonance from BIC facilitated a remarkable transmission change with a small variation in the Fermi level. The Q of the proposed structure is approximate ~ 2 × 10^5^, and this value is about ten times higher than previous reported graphene-based resonators. In addition, the Q can increase easily by tuning the incident angle. The proposed modulators could simultaneously support a high modulation depth and low insertion loss: MD_ISLG_ = 0.9972, IL_ISLG_ = 0.0034, MD_ISLG_ = 0.9889, and IL_ISLG_ = 0.011. The effect of graphene mobility on the performance of the modulators was also investigated. Although low graphene mobility (*μ* = 0.1 m^2^/Vs) deteriorated the efficiency of the modulator, an outstanding efficiency could be maintained for standard quality graphene (*μ* > 0.5 m^2^/Vs). These promising features are likely to generate new avenues for graphene-based optical devices for free-space applications.

## Methods

The permittivity of graphene was calculated using the Kubo formula, assuming a graphene thickness of 0.34 nm and Fermi velocity of 10^6^ m/s. The reflection spectrum of the photonic crystal structure without graphene as a function of the incident angle was calculated using rigorous coupled-wave analysis (RCWA), and the transmission spectra of the modulators were calculated using the COMSOL Multiphysics software. Periodic boundary condition was used for the calculations, and a sufficiently small mesh size was used for accurate calculations.
